# A protective nesting association with native species counteracts biotic resistance for the spread of an invasive parakeet from urban into rural habitats

**DOI:** 10.1186/s12983-020-00360-2

**Published:** 2020-05-07

**Authors:** Dailos Hernández-Brito, Guillermo Blanco, José L. Tella, Martina Carrete

**Affiliations:** 1 Department of Conservation Biology, Estación Biológica de Doñana (CSIC), Avda. Américo Vespucio, 41092 Sevilla, Spain; 2 Department of Evolutionary Ecology, Museo Nacional de Ciencias Naturales (CSIC), C/ José Gutiérrez Abascal 2, 28006 Madrid, Spain; 3 Department of Physical, Chemical and Natural Systems, University Pablo de Olavide, Ctra. de Utrera km. 1, 41013 Sevilla, Spain

**Keywords:** Biological invasions, Biotic resistance, Monk parakeet, Predation pressure, White stork, Commensalism, Facilitation

## Abstract

**Background:**

Non-native species are often introduced in cities, where they take advantage of microclimatic conditions, resources provided by humans, and competitor/predator release to establish and proliferate. However, native communities in the surrounding rural or natural areas usually halt their spread through biotic resistance, mainly via top-down regulative processes (predation pressure). Here, we show an unusual commensal interaction between exotic and native bird species that favours the spread of the former from urban to rural habitats.

**Results:**

We show how Monk parakeets *Myiopsitta monachus*, an invasive species often introduced in cities worldwide, associated for breeding with a much larger, native species (the white stork *Ciconia ciconia*) to reduce predation risk in central Spain, thus allowing their colonization of rural areas. Parakeets selected stork nests close to conspecifics and where breeding raptors were less abundant. Parakeets always flushed when raptors approached their nests when breeding alone, but stayed at their nests when breeding in association with storks. Moreover, when storks abandoned a nest, parakeets abandoned it in the following year, suggesting that storks actually confer protection against predators.

**Conclusions:**

Our results show how a protective-nesting association between invasive and native species can counteract biotic resistance to allow the spread of an invasive species across non-urban habitats, where they may become crop pests. Monk parakeet populations are now growing exponentially in several cities in several Mediterranean countries, where they coexist with white storks. Therefore, management plans should consider this risk of spread into rural areas and favour native predators as potential biological controllers.

## Background

Cities are often the first point of introduction for many alien species [75], some of which can take advantage of microclimatic conditions [36], resources provided by humans [55], or competitor or/and predator release [4, 50] to establish and proliferate. Thus, cities have become hotspots of invasive species [47], which can secondarily spread into nearby, rural landscapes (e.g. [3, 5, 60, 112]). However, native communities in the areas surrounding urban cores usually halt the spread of non-native species through biotic resistance [34], which may arise through competition [45, 101, 111] but also via top-down regulative processes such as predation [26, 40, 85, 87]).

Several studies have shown that predators can limit local population size or habitat use of invaders [26, 30, 85, 87, 104], although results are not conclusive. For instance, in some systems, native predators prey minimally on invaders [108] while in others, even if predators heavily prey on an invader, they have little overall influence on its success due to life history characteristics that compensate for high predation rates [85]. In other cases, the variable influence of predators on invasion success may be due to facilitative interactions among invasive and native species [89, 115] or to habitat heterogeneity, which can create refuges where invasive species experience relaxed predation [26, 35, 49, 57, 59, 64].

Interspecific protective associations are both facilitative interactions and a form of refuge where individuals of one species exploit the antipredatory behaviour of another relatively aggressive species to reduce predation risk [82]. These associations allow individuals to occupy habitats that are otherwise unsuitable because of the negative effects of predation [79, 81], increasing the realised niche of a species [22]. Despite the existence of several examples about facilitative interactions favouring invasive plants and invertebrates [6, 74, 86], as well as positive effects of habitat refugees on invasion success [76, 114], to our knowledge, no studies are testing the effect of interspecific protective interactions on invasion success.

Here, we explore the role of interspecific protective nesting interactions in facilitating the spread of an urban invasive avian species, the monk parakeet (*Myiopsitta monachus*), into neighbouring rural areas. The monk parakeet is one of the most widespread avian invaders, with invasive populations mainly in North America and Western Europe, but also in Asia, Africa and some oceanic islands [103]. This species has spread due to the international trade of millions of wild-caught parakeets from their native South American range [29, 31, 43] to pet shops and homes across the globe. Posterior accidental escapes or releases have founded several invasive populations in urban habitats [2, 3, 53, 102, 109]. The fact that most of the non-native populations are urban may be explained by predation release, which allows a higher breeding success in their invaded urbanised habitats than in their native ranges, as was also shown for the highly invasive rose-ringed parakeet *Psittacula krameri* [97]. In fact, recent work has demonstrated that the breeding success of monk parakeets is twice as high in a Spanish city than in its native range [95]. Other hypotheses, such as competition or parasite release, seem unlike to explain the high success of monk parakeets in urban areas. Contrarily to the secondary cavity-nesting rose-ringed parakeet, which competes with native species for nest holes [52], monk parakeets are unique among parrots as they build their own nests using wood sticks on trees and artificial substrates such as power pylons and roofs [65, 95], thus avoiding competition for nest sites. Regarding parasite release, monk parakeets gain novel parasites from the recipient community in its invaded range while also maintain parasites from its native range [7, 19, 70].

**Fig. 1:**
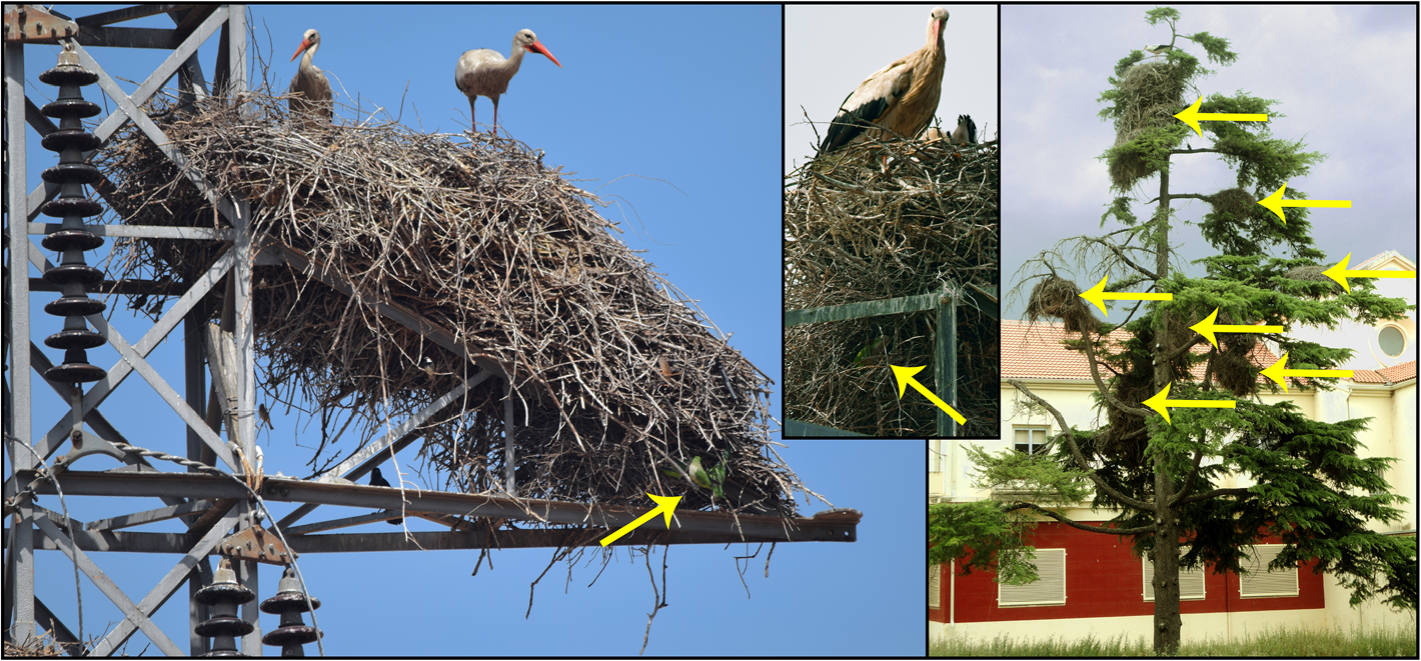
Parakeets and storks. Nests of monk parakeets (yellow arrows) associated with white stork nests. (Photos: D. Hernández-Brito)

Contrasting to their general urban habits in invaded regions, the largest Spanish population of monk parakeets (located in Madrid) has spread into the nearby rural area in recent years, in association with the massive nest structures of white storks *Ciconia ciconia* (Fig. 1). Previous studies show that, in its native range, monk parakeets can also build their nests associated with the nests of other stork species such as jabirus *Jabiru mycteria* [23]. In the surroundings of Madrid, parakeets build their nests associated with nests occupied by breeding white storks. Despite parakeets can steal nest material from conspecific neighbours [16, 42], we have never recorded cases of parakeets stealing sticks -to storks, may be due to of differences in the size and ductility of building materials for each species. Although nesting site availability does not explain the use of stork nests, as monk parakeets may use a variety of nesting substrates [65, 95], this association may confer breeding advantages to parakeets [23]. Several small-sized native bird species such as sparrows and starlings are also tolerated by white storks and use their nests as nesting substrates [54]. The large-bodied white stork has extremely low nest predation rates, as learned from long-term breeding monitoring programs in Spain (e.g., [8, 110]), and predation of adults is anecdotal [106]. Therefore, smaller bird species may also associate with white storks to reduce predation risk [15, 56], as it has been demonstrated for other protective nesting associations [82].

Here, we hypothesised that monk parakeets associate with storks not because of structural benefits but to obtain protection against predation, thus allowing their spread into rural habitats despite the existence of a large predator community. To test this hypothesis, we first compared the probability of parakeet-stork associations between rural and urban habitats, predicting that they should be more frequent in the former, where predators are more abundant than in the latter. We then assessed whether these associations occur at random, or if they can be explained by a combination of biotic and abiotic factors such as the proximity of the stork nest to another parakeet colony (conspecific density), the type of substrate (pylons, trees, or roofs), or the density of predators in the surroundings (predation risk). We predicted that besides proximity to conspecifics, parakeets should breed preferentially in stork nests sited in pylons (thus reducing predation risk at the nest) in areas with a low density of aerial predators (thus reducing predation risk while foraging). The low density of predators in the surrounding of a nest ensures safe areas for parakeets to perform basic activities such as foraging. Nests in pylons, contrary to those located in trees or on roofs, are not accessible to mammalian or reptilian predators [15], so the antipredatory effect of nesting with a stork is focused on aerial predators (i.e., raptors) and, thus, maximised. Finally, to discard the potential benefits derived from the nest structure per se, we evaluated the effect of nest abandonment by storks on the subsequent nest abandonment by parakeets. We predict that parakeets should abandon their nests after stork abandonment due to the disappearance of its protective effect against predators; otherwise, parakeets could associate with storks to simply take advantage of their nest structures. Complementarily, we compared the behaviour of parakeets toward approaching avian predators when breeding in association with storks or not. We predicted that parakeets should flush more frequently from raptors when breeding alone than when breeding with storks, where they can take advantage of the presence of the protective species, which can deter raptor attacks.

## Results

### Protective nest associations

**Fig. 2:**
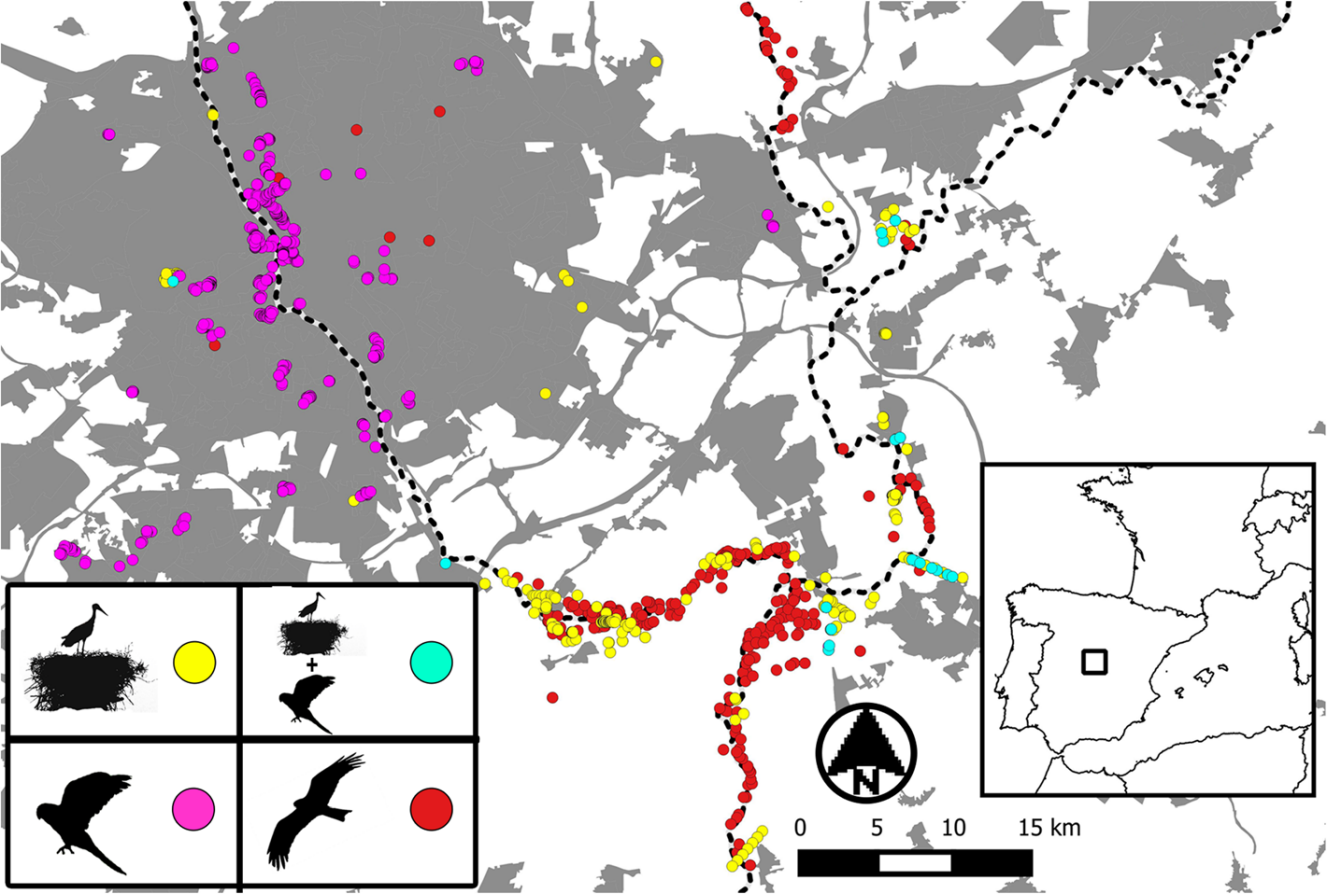
Study area. Urban (dark grey) and surrounding rural (white) areas of Madrid Metropolitan area (40° 21′ 03.1“ N, 3° 30’ 06.1” W). Different coloured points show the location of nests of raptors (red), storks (yellow), and parakeets associated (blue) or not associated (purple) with storks. Black dashed lines are rivers

We recorded more than 900 monk parakeet nests, most of them located in the urban habitat, while avian predators were much more abundant in rural habitats (Fig. 2; Table 1). Parakeets nesting in rural habitats were rare and mainly associated with storks (97.06% of parakeet nests associated with storks in 2014, *n* = 34; 73.53% of parakeet nests associated with storks in 2015, n = 34), an association that was near completely absent among urban parakeets (habitat (urban): estimate: -2.30; 95% CI: − 2.88 - -1.73). Importantly, parakeets bred mainly associated with stork nests located in pylons (70 and 100% of associated nests in 2014 and 2015 were in pylons, Table 1). However, models run for 2014 and 2015 show that the probability of parakeet-stork association was only related to habitat (i.e., more likely in rural than in urban areas; Table 2).

**Table 1:** Abundance of species in the study area. Number of nests of Monk parakeets*,* white storks and avian predators in urban and rural areas. The type of substrate (i.e., pylon, tree or roof) is indicated for Monk parakeet and white stork nests

Species	2014		2015	
	Urban	Rural	Urban	Rural
Monk parakeets *Myiopsitta monachus*	867	34	890	34
Associated with storks				
Pylons	0	23	0	25
Trees	9	10	9	0
Roofs	0	0	0	0
Not associated with storks				
Pylons	2	0	2	0
Trees	856	1	879	9
Roofs	0	0	0	0
White storks *Ciconia ciconia*	41	466	47	440
Pylons	6	233	7	209
Trees	32	217	37	216
Roofs	3	16	3	15
Black kites *Milvus migrans*	0	239	0	244
Common buzzards *Buteo buteo*	0	15	0	14
Booted eagles *Hieraaetus pennatus*	0	5	0	4
Northern goshawks *Accipiter gentilis*	0	3	0	3
Red kites *Milvus milvus*	0	1	0	1
Peregrine falcon *Falco peregrinus*	6	0	6	0

**Table 2:** Factors affecting the probability of association between parakeets and storks. Relative importance of habitat (urban and rural) and type of substrate (pylon, tree or roof) on the probability of protective nesting associations between monk parakeets *Myiopsitta monachus* and white storks *Ciconia ciconia*. Estimates and 95% confidence intervals (2.5 and 97.5%) were assessed after model averaging (ΔAIC ≤2). We considered that a given variable has no, weak or strong support when the 95% confidence interval strongly overlapped zero, barely overlapped zero (*), or did not overlap zero (**), respectively. *k*: number of parameters. AICc: Akaike Information Criterion corrected for small sample sizes. ΔAICc: difference between the AICc of model *i* and that of the best-supported model (i.e. the model with the lowest AICc); w: Akaike weights. R^2^: measure of how well the model explains the data

Model	k	AICc	ΔAICc	Weight	Variables	Estimate	2.5%	97.5%	
substrate*habitat + year	5	234.58	0.00	0.58	substrate (pylon)	18.66	− 1814.86	1852.17	
substrate*habitat	4	236.40	1.81	0.24	habitat (urban)	−4.59	−5.61	−3.58	**
substrate + habitat + year	4	237.66	3.08	0.13	year (2015)	−0.85	−1.73	0.03	*
substrate + habitat	3	239.22	4.64	0.06	habitat (urban)*substrate (pylon)	−32.65	− 6642.70	6577.41	
habitat*year	4	257.07	22.49	0.00					
habitat + year	3	260.93	26.35	0.00					
habitat	2	261.53	26.95	0.00					
substrate + year	3	318.70	84.12	0.00					
substrate	2	320.07	85.48	0.00					
	1	633.94	399.36	0.00					
year	2	634.84	400.26	0.00					

R^2^ = 0.70

**Fig. 3:**
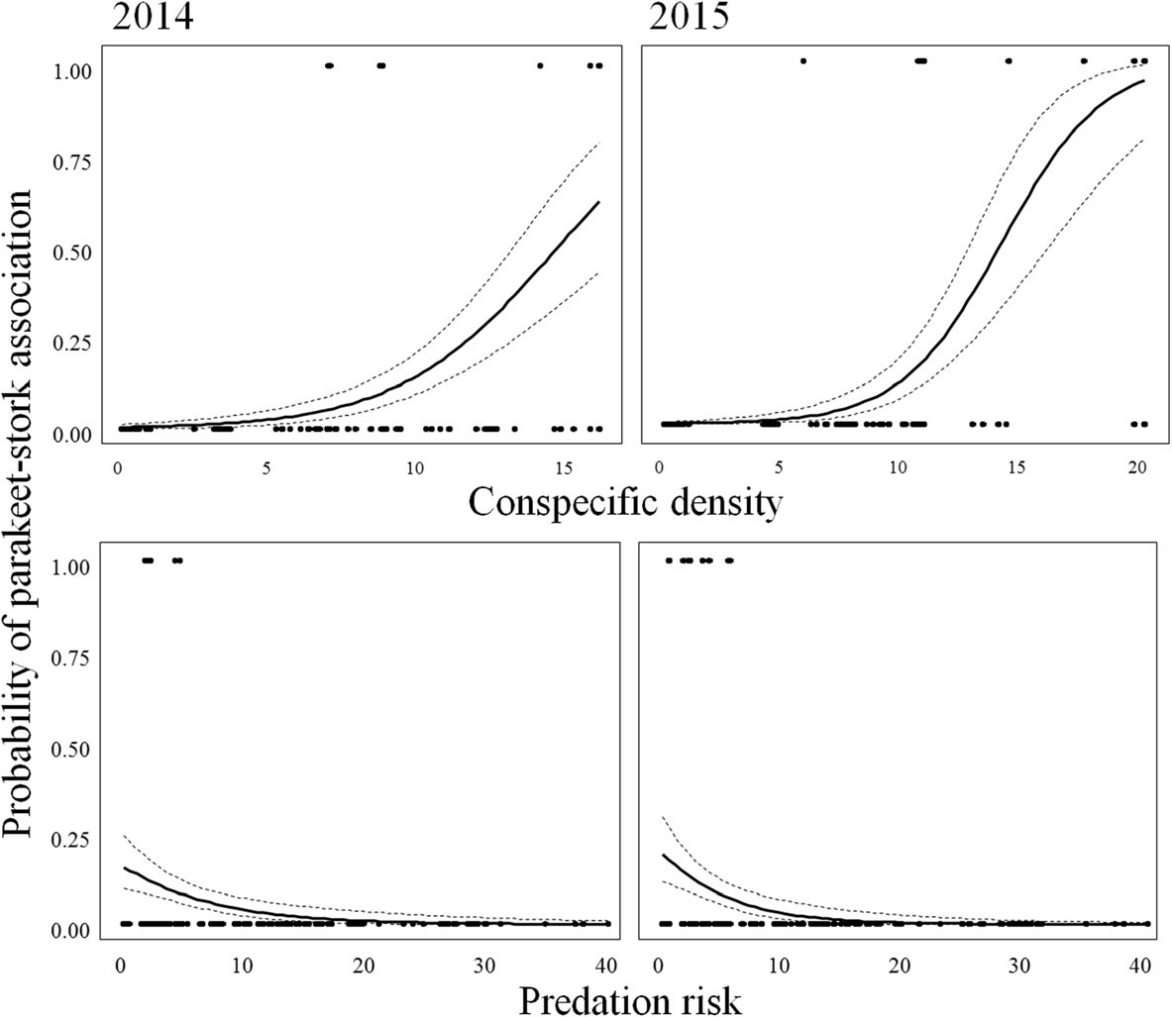
Probability of parakeet-stork association in rural habitats. Parakeets select rural stork nests located farther from predators (aggregation of predators) and surrounded by larger densities of conspecifics (aggregation of parakeets). Estimates (solid lines), confidence intervals (dashed lines) and raw data (black dots) are shown for 2014 and 2015

Within rural habitats, stork nests were not used at random, and parakeets selected, among those available, stork nests located in areas where conspecifics were more abundant and predation risk was lower (Table 3; Fig. 3). It is worth noting that the correlation between conspecific density and predation risk increased from 2014 to 2015 (− 0.38 and − 0.48, respectively), so predation risk received weaker support in models obtained for the second year. However, when conspecific density was excluded from models, predation risk was strongly related to stork nests also used in 2015 (estimate: -1.34, 95% CI: − 2.60 - -0.47).

**Table 3:** Factors affecting the probability of association between parakeets and storks in rural areas. Relative importance of predation risk, conspecific aggregation and substrate on the probability of protective nesting associations between Monk parakeets *Myiopsitta monachus* and white storks *Ciconia ciconia*. Estimates and 95% confidence intervals (2.5 and 97.5%) were assessed after model averaging (ΔAIC ≤2). We considered that a given variable has no, weak or strong support when the 95% confidence interval strongly overlapped zero, barely overlapped zero (*), or did not overlap zero (**), respectively. Models were run separately for 2014 and 2015. *k*: number of parameters. AICc: Akaike Information Criterion corrected for small sample sizes. ΔAICc: difference between the AICc of model *i* and that of the best-supported model (i.e. the model with the lowest AICc); w: Akaike weights. R^2^: measure of how well the model explains the data

Model 2014	k	AICc	∆AICc	weight	Variables	Estimate	2.50%	97.50%	
predation risk + conspecific density	3	107.56	0.00	0.67	predation risk	−3.70	−5.47	−1.94	**
predation risk + conspecific density + substrate	5	108.94	1.38	0.33	conspecific density	2.69	1.56	3.81	**
conspecific density + substrate	4	128.75	21.19	0.00	substrate (pylon)	15.22	− 2533.35	2563.80	
conspecific density	2	133.87	26.31	0.00	substrate (tree)	16.21	− 2532.36	2564.79	
predation risk + substrate	4	173.90	66.34	0.00					
predation risk	2	180.08	72.52	0.00					
substrate	3	183.32	75.76	0.00					
	1	202.61	95.04	0.00					
**Model 2015**	**k**	**AICc**	**∆AICc**	**weight**	**Variables**	**Estimate**	**2.50%**	**97.50%**	
conspecific density + substrate	4	101.07	0.00	0.36	conspecific density	2.02	1.19	2.84	**
conspecific density	2	101.39	0.32	0.31	substrate (pylon)	18.40	−13,219.31	13,256.10	
predation risk + conspecific density	3	102.34	1.26	0.19	substrate (tree)	1.63	−13,742.57	13,745.84	
predation risk + conspecific density + substrate	5	102.87	1.80	0.15	predation risk	−0.75	−2.75	1.25	
predation risk + substrate	4	149.78	48.71	0.00					
substrate	3	159.11	58.04	0.00					
predation risk	2	164.26	63.18	0.00					
	1	193.96	92.88	0.00					

2014: R^2^ = 0.39

2015: R^2^ = 0.40

### Probability of nest abandonment by parakeets

From 2014 to 2015, 44% of the 34 rural parakeet nests were abandoned, while all urban parakeet nests remained active. This high rate of abandonment recorded among rural nests was strongly explained by the abandonment of the nest by the stork (Table 4). Substrate, conspecific density and predation risk were weakly supported, with nests located in pylons, far from conspecifics and in areas with a low density of predators being more prone to abandonment when they were abandoned by storks.

**Table 4:** Factors affecting the probability of nest abandonment by parakeets between 2014 and 2015. Estimates and 95% confidence intervals (2.5 and 97.5%) were assessed after model averaging (ΔAIC ≤2). We considered that a given variable has no, weak or strong support when the 95% confidence interval strongly overlapped zero, barely overlapped zero (*), or did not overlap zero (**), respectively. Models were run separately for 2014 and 2015. *k*: number of parameters. AICc: Akaike Information Criterion corrected for small sample sizes. ΔAICc: difference between the AICc of model *i* and that of the best-supported model (i.e. the model with the lowest AICc); w: Akaike weights. R^2^: measure of how well the model explains the data

Model	k	AICc	ΔAICc	weight	Variables	Estimate	2.5%	97.5%	
abandon stork + conspecific density	3	31.88	0	0.37	abandon stork	3.83	0.82	6.85	**
abandon stork	2	32.95	1.07	0.22	conspecific density	−1.03	−2.31	0.25	*
abandon stork + substrate	3	33.01	1.13	0.21	substrate (pylon)	1.81	−0.81	4.43	*
abandon stork + predation risk	3	33.23	1.35	0.19	predation risk	−0.87	−2.20	0.47	*
predation risk + conspecific density	3	41.39	9.52	0.00					
Null	1	41.42	9.55	0.00					
conspecific density + substrate	3	42.19	10.32	0.00					
Substrate	2	42.89	11.02	0.00					
predation risk	2	42.95	11.08	0.00					
conspecific density	2	42.96	11.08	0.00					
predation risk + conspecific density + substrate	4	43.43	11.56	0.00					
predation risk + substrate	3	45.27	13.39	0.00					

R^2^ = 0.45

### Interactions between monk parakeets and predators

**Fig. 4:**
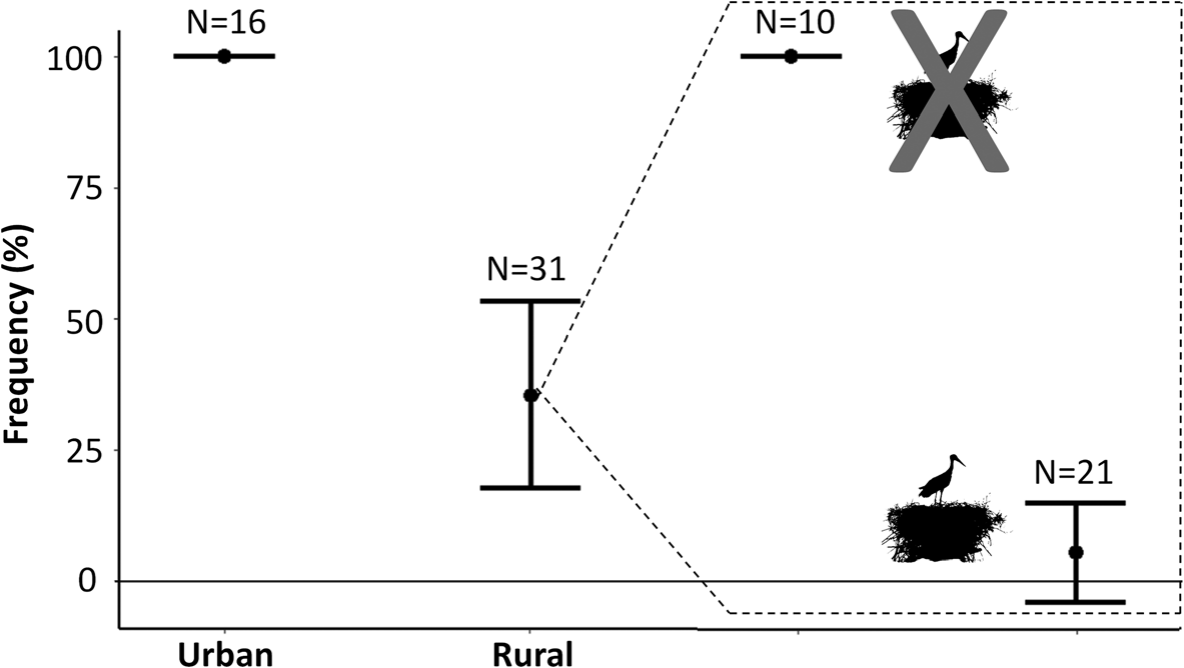
Antipredatory response of monk parakeets. Frequency of observations (mean and 95% CI) in which nesting monk parakeets flushed when approached by a predator (raptor) in urban and rural habitats. For rural individuals, the frequency of birds flushing is separately shown for nests associated or not associated with storks

We recorded 47 instances in which three different raptor species (the black kite, the booted eagle, and the common buzzard) closely approached parakeet nests, most of them in rural habitats (66%). The number of parakeets present during these intrusions ranged between 1 and 50 (median = 8.7). Parakeets usually flew when raptors approached (57.4%), although in 36.2% of the cases they stayed in the nests. Parakeets attacked the intruding raptor (mobbing) only in three cases (6.4%), all of them in rural nests associated with stork nests. Given the low number of mobbing events, we considered the proportion of flushing events against mobbing and staying (pooled together) between habitats. Parakeets from urban nests flushed in 100% of the raptor intrusions while rural ones only flushed in 31.5% of the events (Fig. 4). This behavioural difference is statistically significant (*χ*^*2*^ = 878.28, df = 2, *p <* 0.001), even when controlling for the potential effects of the raptor species (*χ*^*2*^ = 2.29, df = 2, *p* = 0.318) and the number of parakeets involved (*χ*^*2*^ = 1.70, df = 1, *p* = 0.192). Interestingly, within the rural parakeet population, individuals in nests not associated with storks behaved similarly to urban ones (i.e, they flushed in 100% of the raptor intrusions), while parakeets associated with white storks only flushed in 4.8% of the instances (Fig. 4). Again, this behavioural difference is statistically significant (*χ*^*2*^ = 47.56, df = 2, *p* < 0.001), even when controlling for the potential effects of the raptor species (*χ*^*2*^ = 2.21, df = 2, *p* = 0.331) and the number of parakeets involved (*χ*^*2*^ = 2.98, df = 1, *p* = 0.084).

## Discussion

Most hypotheses dealing with biological invasions focus on factors that increase invasion success while very few focus on aspects that inhibit them [34]. Among the later, studies about interactions between invasive species and their recipient communities have long focused on biotic resistance, mainly through competition and predation [18]. However, some positive interactions with native species may also arise and assist invasive species in establishing populations in novel areas, increasing their potential niche and thus, invasive potential [22]. In this way, recent studies have shown the important role played by mutualisms in enhancing invasions of many exotic plants and invertebrates [107]. However, to our knowledge, there is no published information on protective associations favouring vertebrate invasions. Here, we show how nesting in association with a protective, much larger native species may favour the colonisation of natural habitats by a species typically restricted to urban areas in its invasive range, the monk parakeet. This example, representing another form of ecological facilitation, namely commensalism (i.e., a species interaction in which one species benefits and the other is unaffected), has been more rarely documented [88] and can also be important in assisting invasive species.

Nesting associations between monk parakeets and other stork species have been previously described in South America (the native range of parakeets). However, the authors argued that the main benefit of these assemblages is related to structural safety, noting its potential antipredatory role as a secondary advantage [23]. In our study area, results suggest that the principal explanation for parakeet-stork associations is the deterrence of predators, not the facilitation of nest building. First, records of nesting associations with storks are rare in urban areas, which is unexpected if the main reward is structural benefits. Conversely, all nesting associations occurred in rural areas, where predators were much more abundant. Second, the abandonment of a stork nest by parakeets was related to its previous abandonment by storks, in line with expectations if the protective association allows parakeets to colonise otherwise unsuitable areas in terms of predation risk. Third, when comparing the behavioural responses of parakeets toward predators in rural and urban areas, we found that urban birds, which are not associated with storks, always flushed when approached by a predator. The same behaviour was observed among rural parakeets not associated with storks, while almost none of the rural parakeets nesting with storks flushed when a raptor approached their nests. Fourth, contrary to Burger and Gochfeld [23], we considered as associated nests not only nests sharing the structure of the stork nests but also those located within a close radius of the stork nests, thus without direct structural benefits. We can assert that this nesting association, rather than facilitating nesting substrate, offers antipredatory protection that allows the spread of the invader from urban into rural areas.

Although protective nesting associations with storks may overcome the biotic resistance offered by the rural predator community, the importance of predation risk in the distribution of monk parakeets was evident when we analysed the location of these protective associations within the distribution of rural stork nests. From all stork nests available, parakeets tended to associate with those located farther away from predators and surrounded by larger densities of conspecifics. Although storks provide a protective umbrella to parakeets, this protection is restricted to the nearby surroundings of the nest site [82]. Thus, breeding farther from predators may allow parakeets to perform their daily activities such as foraging in areas with lower predation risk. Accordingly, the importance of breeding close to conspecifics can also be explained in terms of reducing predation risk [21, 72, 113], through both cooperative defence and dilution effects [9, 10, 116].

Non-native species tend to proliferate more frequently in human-altered habitats compared to less altered ones [93]. Some bird species such as parrots fit well to this pattern, as they are not only more frequent and abundant in such environments, mainly urbanised ones [3, 28], but because many of them seem to be unable to expand to more pristine habitats despite their well-developed flying abilities [32, 99]. Although the reasons are unclear, some authors have proposed that the higher biotic resistance often recorded in more natural communities can preclude the colonisation of more natural habitats by exotic species [41, 44, 99]. In this sense, predators can exert strong top-down regulative processes on prey populations, mainly when populations are small [73] as occurs during the first stages of the invasion process, when populations of invasive species are still incipient and, thus, particularly vulnerable to predation [33]. For monk parakeets, predation of eggs, chicks and adults in the nests is the most common cause of breeding failure and mortality during the breeding season [42, 62, 63, 72]. Thus, although the species expanded into the rural habitats of the study area in the 1990s (four breeding colonies) when the predator community was much less abundant [14], they failed to successfully establish likely due to the progressive recovery of the predator community since the early 2000s onward (G. Blanco and Ó. Frías, unpubl. data). Spanò and Truffi [100] also recorded the extinction of an emergent monk parakeet population in Italy in 1948 due to constant nest predation by rats, supporting the role of predation in the establishment success of this invasive species.

The management of invasive species in cities is controversial, mainly when the best management option, from an ecological perspective, is the eradication of charismatic species. Ethical conflicts are particularly exacerbated when these species are aesthetically appealing mammals or birds that have become the most visible, non-domesticated animals present in public areas [11]. In those cases, for social and pragmatic reasons, some authors have suggested that these species should be accepted as part of the urban ecosystem if their risk of spreading toward natural areas is reduced [48]. However, the limited capacity of some non-native species to spread from urban habitats may only represent a transient stage related to lag phases, which are expected when evolutionary change -including the evolution of invasive life-history characteristics, the purging of genetic load responsible for inbreeding depression or the evolution of adaptations to the new habitat- is an important part of the colonisation process [38, 58, 90]. Spread lag phases may take years or decades, can vary among species and populations of the same species subject to different ecological conditions [1] and are highly unpredictable [37]. Therefore, it is not prudent to assume that exotic species that have been observed in urban areas for a long period will remain strictly urban in the future. In fact, the distribution of monk parakeets invading Israel has increased and shifted from predominantly urban areas to agricultural landscapes in less than two decades [77]. This may have been facilitated by the long-term decline and poor conservation status of raptors in that country [117]. Thus, accepting invasive species as part of the urban ecosystem may sometimes result in their spread into adjacent rural landscapes, where they could have different, often unknown impacts [17, 69], such as crop damage in the case of monk parakeets [77, 94].

The monk parakeet was first introduced in Spain in 1976, increasing its distribution since then at a rate of 8.14 grid cells (5 × 5 km) per year [3]. However, it was not until recently that the Spanish population began to grow exponentially, increasing from c. 6000 individuals in 2010 to c. 20,000 in 2015 distributed across > 130 urban populations [68]. Therefore, it seems that the species has overcome the lag phase, and the protective nesting association facilitating its spread outside of cities, rather than anecdotal, may be occurring in other areas. Predictive models indicate that there is still plenty of suitable habitat for the species [68, 71]. Furthermore, white storks are widely distributed across Spain (with > 33,000 nests in 2004 [67]) and populations are rapidly increasing thanks to the use of human-related food subsidies (invasive American crayfish and rubbish dumps [13, 92, 105]. In fact, in recent years, monk parakeets also spread from another Spanish city (Zaragoza, 270 km distant to Madrid), by nesting in stork nests (J.L. Tella obs. Pers.), but such a spread was halted by the responsible authorities by shooting the whole parakeet population. Therefore, the risk of monk parakeet expansion from urban habitats, thanks to the widespread distribution of white storks, should be considered when designing management strategies for this highly invasive species, which is growing exponentially in Mediterranean countries [78], including Spain, Portugal, France, Italy, Greece, Morocco, and Israel, where the two species coexist [51, 78]. Management actions could be required in the case rural populations of monk parakeets would cause significant impacts [78], although these actions usually show low support from the society [96], even lower when dealing with charismatic species such as parakeets [39]. Consequently, more research and awareness campaigns are necessary not only to know the actual magnitude of the impacts derived from invasive species [12] but also to make management actions effective. In our case, actions only focus on the avoidance of monk parakeets nesting in white stork nests may not be efficient. On the one hand, anti-nesting devices installed in pylons for white storks do not prevent their nesting, even after great management efforts [61]. On the other hand, nest removal would be not efficient because both species show strong fidelity to their nesting substrates and often rebuild their nests very soon [24, 80, 98, 110]. Contrarily, actions aimed to improve predator populations as biological controllers, should be effective to halt the spread of this species into rural areas in the long term.

## Conclusions

This study assesses the nesting association between an invasive bird, the monk parakeet, and a native bird species, the white stork, showing a commensalism relationship in which parakeet colonies associated with stork nests benefit from the effective antipredatory defence of storks. This association was more likely in rural areas, where predation pressure is higher than in nearby urban ones, assisting thus the spread of monk parakeets across the rural environment. Moreover, the abandonment of parakeet colonies after the previous nest abandonment of associated storks, as well as their different behavioural reactions against raptors when associated with storks, suggest that parakeets have a strong dependence of their hosts. However, this protective association is limited as parakeet colonies also avoided high densities of breeding raptors in the study area. Without the facilitation provided by storks, the biotic resistance from the raptor community prevents the invasion success of parakeets. Future studies are needed to assess the complexity of interactions between invasive species and the recipient community, which may be fundamental to develop effective management plans against biological invasions.

## Methods

### Study area and fieldwork

The study was carried out in an extensive area including the city of Madrid and its surrounding rural habitats along the Manzanares and Jarama rivers (Fig. 2), an area mostly devoted to irrigated, intensive agriculture (mainly maize and vegetables) and gravel extraction. In this area, raptors nest mostly in riparian forests, while white storks nest in the same forest as well as on electric pylons and building roofs [13, 14].

During the breeding seasons (April–August) of 2014 and 2015, the study area was repeatedly visited to GPS-locate all nests of parakeets and storks present in the urban and rural habitats (Fig. 2), also recording the type of substrate in which the nests were located (tree, pylon or roof). Moreover, we monitored the community of medium-sized raptor species present in the study area, including the black kite *Milvus migrans*, the booted eagle *Hieraaetus pennatus*, the common buzzard *Buteo buteo,* the northern goshawk *Accipiter gentilis*, the red kite *Milvus milvus* and the peregrine falcon *Falco peregrinus*. Predation of monk parakeets by raptors has been recorded in its invaded range [20, 24, 27, 84], as well in our study area, such as peregrine falcon [91] and golden eagle *Aquila chrysaetos* (E. Navarro pers. comm.). We recorded remains of monk parakeets in several nests of black kites, booted eagles and peregrine falcons and observed their hunting attempts on flying parakeets. The other two raptor species are of similar size and behaviour, and also include birds in their diets [46]. Thus, we considered the five raptor species as potential predators. We did not find evidence for other bird species, such as corvids, preying upon monk parakeets or their nests.

For storks and raptors, each nest corresponds to a single breeding pair, whereas for parakeets each nest can house from one to several breeding pairs accommodated in different chambers (range: 1–35 active chambers). A parakeet nest was classified as being associated with a stork if they shared the same nesting substrate, if the parakeet nest was located on the same structure (i.e., the same tree or electricity pylon) as the stork nest, or if the parakeet nest was within a radius of 15 m of a stork nest (Fig. 1). Although mammals and snakes can predate on parakeets, they were not considered in this study as we do not have accurate information about their distribution and abundance. However, their potential impact on parakeets is discussed based on nest substrate (see previous).

### Interactions between parakeets and raptors

We recorded the responses of parakeets towards intruding raptors (i.e., raptors flying within less than 15 m of an active parakeet nest) across the study area, following previous work conducted on a similar species, the rose-ringed parakeet (*Psittacula krameri* [52];). Parakeet responses were classified as stay (i.e., when parakeets stayed in their nests, showing no sign of fear toward the raptor), mobbing (i.e., when parakeets flew to attack the approaching raptor), or flush (i.e., when parakeets flew away from the raptor).

### Statistical analyses

We used Generalised Linear Models to test if parakeet-stork associations were more likely in rural than in urban areas (logistic link functions, binomial error distributions) by considering all parakeet nests present in the study area and including the habitat where they were located (i.e., urban or rural) as an explanatory variable. We then evaluated if parakeets used rural stork nests (probability of parakeet-stork association considering all stork nests present in the rural areas; logistic link functions, binomial error distributions) based on a combination of conspecific density, type of substrate and predation risk. Conspecific density was obtained as the relative position of each parakeet nest within the parakeet population. We used the formula *Σexp(−d*_*ij*_)*A, where *d*_*ij*_ is the linear distance between each parakeet nest *i* and all parakeet nests *j*, and *A* is the number of chambers per nest *j* [66]. Higher values of this index point to a higher density of conspecifics around a selected location. Predation risk was assessed by using the aggregation of raptors nests as a proxy, calculated using the same index explained previously (note that here *A* always equals 1). Models for the probability of parakeet-stork associations were separately run for 2014 and 2015 because of convergence problems when using generalised linear mixed models and nest as a random term. Finally, we related the probability of nest abandonment by rural parakeets with nest abandonment by the stork (independent variable), considering if colonies occupied in 2014 remained occupied in 2015 (logistic link functions, binomial error distributions). All continuous variables were included in their linear and quadratic forms and standardised before modelling. Model selection was performed using the Akaike Information Criterion corrected for small sample sizes, AICc [25]). Within each set of models (which includes the null model), we calculated the ΔAICc*i* (as the difference between the AICc of model *i* and that of the best model) and the weight (*w*) of each model. Models within 2 AICc units of the best one were considered as alternatives and used to perform model averaging (MuMIn package). We considered that a given effect received no, weak or strong statistical support when the 95% confidence interval (CI) strongly overlapped with zero, barely overlapped with zero, or did not overlap with zero, respectively. Statistical analyses were conducted in R 3.1.2 [83].

Differences in behavioural responses of parakeets toward approaching raptors were compared among habitats (urban or rural) and between nests associated or not associated with storks using generalised linear models (multinomial error distribution). We included the number of parakeets present in each raptor intrusion and the interacting raptor species as covariates to control for their potential effects.

## Data Availability

Data are sensitive information about the location of nests, especially the raptors, being thus essential for their conservation. Consequently, they are only available on request of authors. The rest of the raw data are provided in the body of the article.
