# Correction: A protective nesting association with native species counteracts biotic resistance for the spread of an invasive parakeet from urban into rural habitats

**DOI:** 10.1186/s12983-023-00490-3

**Published:** 2023-04-06

**Authors:** Dailos Hernández-Brito, Guillermo Blanco, José L. Tella, Martina Carrete

**Affiliations:** 1 Department of Conservation Biology, Estación Biológica de Doñana (CSIC), Avda. Américo Vespucio, 41092 Seville, Spain; 2 Department of Evolutionary Ecology, Museo Nacional de Ciencias Naturales (CSIC), C/ José Gutiérrez Abascal 2, 28006 Madrid, Spain; 3 Department of Physical, Chemical and Natural Systems, University Pablo de Olavide, Ctra. de Utrera km. 1, 41013 Seville, Spain

**Correction: Frontiers in Zoology 17, 13 (2020)** 10.1186/s12983-020-00360-2

Following publication of the original article [1], the authors reported that the funding information has to be updated.

The correct funding information should read:

This study was supported by project CGL2015-71378-P MINECO/FEDER, UE, the Severo Ochoa Program (SVP-2014-068732) and Action COST ES1304 (ParrotNet). Logistical and technical support for fieldwork was provided by Doñana ICTS-RBD.

The original article [1] has been updated.
